# Pulmonary thromboembolism: multidisciplinary collaboration or confrontational terrain between specialties?

**DOI:** 10.1186/s42155-024-00506-x

**Published:** 2024-12-18

**Authors:** Sara Lojo-Lendoiro

**Affiliations:** Complexo hospitalario de Ferrol Interventional Radiologist, Radiology Department, Hospital Arquitecto Marcide, Av. da Residencia, S/N, Ferrol, 15405 A Coruña Spain

**Keywords:** Pulmonary thromboembolism, Multidisciplinary collaboration, Interventional radiology, Patient management, Thrombectomy

Pulmonary thromboembolism (PTE) is a highly serious medical condition, which demands speed, diagnostic and therapeutic precision, and, in theory, the interdisciplinary collaboration of cardiologists, interventional radiologists and other specialists. However, far from being an ideal model of working together, the management of PTE has revealed a latent conflict between specialties that does not always prioritise the patient, but is often marked by ego, jockeying for position and an implicit competition to dominate the field.

The dominant narrative in the medical literature lauds the importance of multidisciplinary teams, especially Pulmonary Embolism Response Teams (PERTs), as the key to improving clinical outcomes in PTE. But behind this façade of collaboration lies a complex dynamic that deserves to be critically analyzed. Is the collaboration genuine, or is it a silent battle where specialties seek to consolidate their influence and dominance over the care of a condition that should be exclusively patient-centered?

## Cardiologists vs. interventional radiologists: two conflicting views

The conflict between cardiologists and interventional radiologists in the management of PTE has deep roots. Each specialty approaches PTE from different perspectives, which may complement each other, but often clash in clinical practice.

Cardiologists, with their focus on cardiovascular pathophysiology, tend to focus on hemodynamic risk stratification, assessment of impact on cardiac function and pharmacological management, such as anticoagulation and systemic thrombolysis. Historically, they have led the way in the treatment of PTE due to their expertise in the management of right heart failure and associated complications. However, with the increasing popularity of endovascular techniques, they have begun to feel that their role is being displaced.

On the other hand, interventional radiologists have brought innovative solutions to the field, such as mechanical thrombectomy and catheter-directed thrombolysis [1](CDT), which promise to reduce complications and speed recovery. These techniques, which require advanced imaging skills and minimally invasive procedures, have positioned interventional radiologists as key players in the treatment of intermediate-to-high risk PTE. However, their technical approach is sometimes perceived by cardiologists as isolated from the patient’s overall physician context.

## Ego as a barrier to collaboration

A central factor driving this issue is professional ego. Both cardiologists and interventional radiologists bring significant expertise and experience, but this can lead to competitive dynamics. Rather than recognizing each other’s strengths, they focus on asserting their own relevance within the team, seeking to consolidate influence over PTE management.

This ego-driven dynamics manifests in several ways:

1. **Disputes over leadership**: In many hospitals, there is no clear protocol on who should lead the management of PTE. This leads to unnecessary bickering rather than agile decision-making.
2. **Disagreement about optimal treatment**: While interventional radiologists may advocate invasive interventions, cardiologists may lean towards conservative management, especially in intermediate-risk patients. Rather than reaching an evidence-based consensus, these discussions often reflect differences in approach rather than patient needs.
3. **Lack of mutual respect**: In some cases, collaboration is hampered by biases between specialties, where one party undervalues or questions the expertise of the other.

The impact of these dynamics on the patient cannot be underestimated. Every minute lost in interprofessional disputes increases the risk of serious complications, including death. Effective medical care must prioritize the patient, placing outcomes above personal or professional recognition.

## Overlapping roles and lack of clear protocols

Another major problem is the overlapping of roles. With the advancement of endovascular techniques, the boundaries between the responsibilities of each speciality have become more blurred. In some hospitals, cardiologists have begun to perform mechanical thrombectomy procedures, while interventional radiologists have sometimes taken on traditional clinician roles. This lack of definition not only creates tensions, but also confuses patients, who may receive conflicting messages about their diagnosis and treatment.

Moreover, the absence of unified protocols exacerbates this problem. Although international clinical guidelines, such as those of the ESC (European Society of Cardiology), provide general recommendations, their implementation varies significantly between hospitals. Without clear protocols, decisions are often left to interpersonal relationships between teams, which can lead to inconsistencies in care.

## And the patient?

The biggest loser in this confrontation is undoubtedly the patient. When specialties compete rather than collaborate, time and resources are wasted, and critical decisions are delayed. Patients face not only medical risks, but also a fragmented and confusing experience that can affect their trust in the healthcare system.

Instead of prioritizing patient wellbeing, PTE management often becomes an arena where specialties seek to prove their relevance, hoping to consolidate their dominance in an evolving field. This self-centered approach is not only inefficient, but also ethically questionable.

## What can be done? Proposals for change

1. **Establish clear protocols**: Hospitals should implement specific guidelines that define roles and responsibilities in the management of PTE. This would not only reduce tensions, but also ensure faster and more effective decisions.
2. **Joint training**: Interdisciplinary workshops and simulations can help cardiologists and interventional radiologists better understand each other’s skills and limitations. This mutual understanding is essential to build respectful and collaborative relationships.
3. **Case-led PET scan response teams**: Rather than assigning leadership to a specific specialty, teams should be led based on the needs of the patient. For example, in a low-risk PTE, the cardiologist might lead conservative treatment, while in an intermediate-high risk case, the interventional radiologist should take the lead.
4. **Foster a patient-centered culture**: Hospital leaders should promote a culture where decisions are based on what is best for the patient, not on interprofessional hierarchies.

## Conclusion

The management of PET should be an example of how modern medicine can benefit from interdisciplinary collaboration [2, 3]. However, tensions between cardiologists and interventional radiologists have turned the field into an arena of confrontation where ego and power struggles often overshadow the needs of the patient. If we are to move forward, we must redefine the approach to one that is truly patient-centered, putting aside individual interests and prioritizing teamwork. Only then can we fulfil our ethical and professional responsibility to provide the best possible care to those who need it most.

The opinions expressed in this article are the sole responsibility of the author.

They do not necessarily reflect the institutional position of the scientific associations to which I belong.

## Data Availability

Not applicable.
